# Trophoblast antigens, fetal blood cell antigens, and the paradox of fetomaternal tolerance

**DOI:** 10.1084/jem.20211515

**Published:** 2022-04-13

**Authors:** Gabrielle Rizzuto, Adrian Erlebacher

**Affiliations:** 1 Department of Pathology, University of California San Francisco, San Francisco, CA; 2 Center for Reproductive Sciences, University of California San Francisco, San Francisco, CA; 3 Biomedical Sciences Program, University of California San Francisco, San Francisco, CA; 4 Department of Laboratory Medicine, University of California San Francisco, San Francisco, CA; 5 Bakar ImmunoX Initiative, University of California San Francisco, San Francisco, CA

## Abstract

The paradox of fetomaternal tolerance has puzzled immunologists and reproductive biologists alike for almost 70 yr. Even the idea that the conceptus evokes a uniformly tolerogenic immune response in the mother is contradicted by the long-appreciated ability of pregnant women to mount robust antibody responses to paternal HLA molecules and RBC alloantigens such as Rh(D). Synthesizing these older observations with more recent work in mice, we discuss how the decision between tolerance or immunity to a given fetoplacental antigen appears to be a function of whether the antigen is trophoblast derived—and thus decorated with immunosuppressive glycans—or fetal blood cell derived.

## Teaser

We review our current understanding of adaptive immune responses to the fetoplacental allograft, emphasizing the divergent responses observed for trophoblast- versus fetal blood cell–derived antigens. We suggest these divergent responses arise from cell type–specific glycosylation programs that impart trophoblast-derived antigens with immunosuppressive properties.

## Introduction

**Figure 1.**
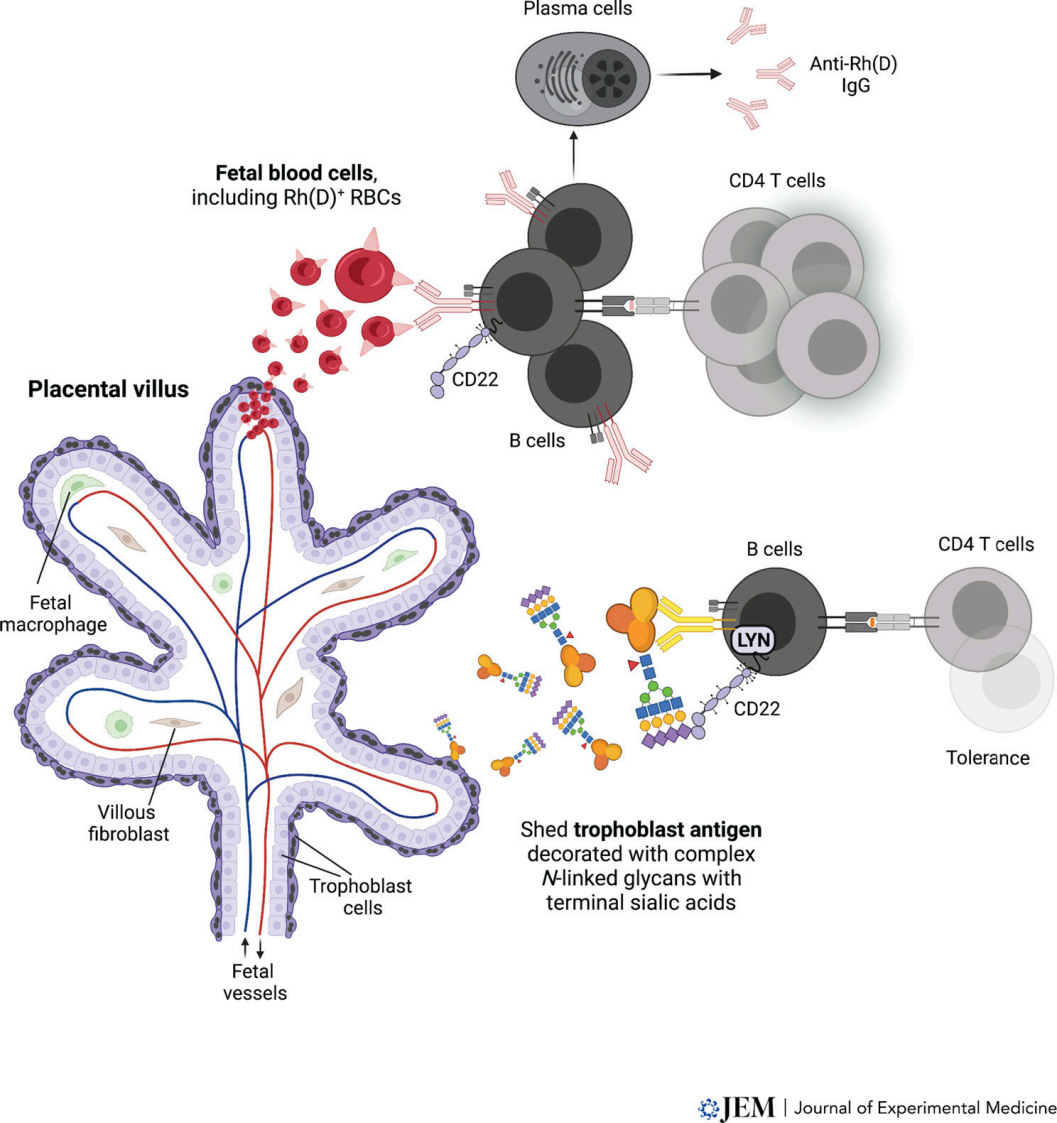
**Divergent responses to fetal blood cell antigens versus trophoblast antigens.** Cartoon representation of a placental villus, showing that this is a trophoblast-lined structure that encases fetal blood vessels. The entire structure is bathed in maternal blood. During pregnancy, maternal immune cells encounter fetal blood cell antigens (upper, illustrating the specific case of Rh(D) antigen) and trophoblast-derived antigens (lower, illustrating a generic trophoblast antigen, modeled by t-mOVA in mice). Upper: Placental microhemorrhage releases Rh(D)^+^ fetal RBCs into the maternal circulation. The RBCs are then recognized by maternal Rh(D)-specific B cells, whose activation and differentiation into plasma cells likely involves cognate interaction with maternal Rh(D)-specific CD4 T helper cells, which presumably have also interacted with maternal DCs (not shown). Because Rh(D) antigen is not sialylated, B cell activation proceeds unimpeded. Ultimately, Rh disease results when the anti-Rh(D) IgG antibodies, transferred across the placenta by the neonatal Fc receptor, bind to the Rh(D)^+^ fetal RBCs and induce their lysis (not depicted). Although less well studied, the maternal antibody response to paternal HLA might occur similarly when placental microhemorrhage allows maternal B cells to encounter fetal white blood cells (not shown). Lower: Sialylated t-mOVA is shed into maternal circulation and is presented to CD4 T cells exclusively by antigen-specific B cells, whose activation is suppressed by concomitant engagement of CD22. CD22 recognizes α2,6-linked sialic acids and requires LYN kinase for signaling. Because B cell activation is suppressed, cognate CD4 T cell activation is also suppressed.

The conceptus, comprised of the placenta and the fetus proper, is not genetically identical to the mother yet fails to induce the rejection response traditionally observed for allogeneic organ transplants. Nearly 70 yr ago, Peter Medawar recognized this apparent paradox and posited three possible explanations: (1) the conceptus is kept physically separate from maternal immune cells; (2) the maternal immune system is generally suppressed; and (3) the conceptus does not express rejection antigens (Medawar, 1953). These ideas have since been largely disqualified for the following reasons. First, the main functional unit of the placenta, i.e., the villous tree in humans and the labyrinth in mice, positions placental epithelial cells (trophoblasts) directly in the maternal bloodstream (see Fig. 1 for the architecture of a human placental villus). Moreover, other populations of trophoblasts invade the uterine lining (the “decidua”), where they can locally interact with maternal immune cells. Second, maternal immune responses to pathogens and experimental foreign antigens are essentially intact across gestation, thus ruling out generalized immunosuppression. Third, trophoblasts secrete proteins into the maternal circulation, and various trophoblast subtypes express a variety of potential alloantigens, including nonclassic MHC class Ib molecules (HLA-E and HLA-G; Apps et al., 2009), oncofetal antigens (Jungbluth et al., 2007), cell type–specific proteins (Moore and Dveksler, 2014), and ubiquitously expressed minor histocompatibility antigens such as H-Y protein (Holland et al., 2012; Linscheid and Petroff, 2013). While human trophoblasts are uniformly negative for HLA class II, HLA-A, and HLA-B, some subtypes express HLA-C (Apps et al., 2009; Hiby et al., 2010; Proll et al., 1999), whose mismatch alone can trigger bone marrow graft failure (Petersdorf et al., 1997). In mice, some trophoblast subtypes express low levels of H-2K/D (Erlebacher et al., 2007; Redline and Lu, 1989), but even transgene-directed expression of allogeneic H-2K at high levels in all trophoblasts does not compromise pregnancy (Rogers et al., 1998; Shomer et al., 1998).

Thus, the paradox of fetomaternal tolerance has become focused on the more refined question of how an allograft that does express rejection antigens and does interact extensively with immune cells in an immune-competent host fails to elicit a traditional rejection response. Here we review our current understanding of systemic maternal B and T cell responses to the fetoplacental allograft, emphasizing the divergence in response apparent for antigens expressed by trophoblasts versus fetal blood cells and the potential role of cell type–specific protein glycosylation in explaining this divergence.

## Maternal B and T cell responses to fetal blood cell antigens

Despite a varied architecture across mammals, the placenta forms an anatomic barrier between maternal and fetal circulation that separates fetal cells of nontrophoblast origin from the maternal immune system. However, in both humans and mice, antigenic quantities of fetal blood cells can enter maternal circulation and trigger maternal alloimmunization (Masson et al., 2013; Porrett, 2018; Urbaniak and Greiss, 2000). In both species, this exposure can occur during delivery, and in humans it can also occur during the third trimester as a result of occult placental microhemorrhage.

The classic example of a situation in which the mother becomes immunized to a fetal blood cell antigen is the clinical condition known as hemolytic disease of the fetus and newborn (HDFN), which is caused by maternal B cells that have reacted to alloantigens expressed by fetal RBCs (Webb and Delaney, 2018). Most cases of alloimmunization occur in women lacking the integral RBC membrane protein Rhesus (Rh)D antigen (Rh(D)^−^ women) who are exposed to Rh(D)^+^ fetal blood during late gestation or delivery. HDFN, or Rh disease, as it is known when Rh(D) is the inciting antigen, can then occur when anti-Rh(D) IgG antibodies are transported across the placenta by the same pathway that provides protective passive immunization to the developing fetus. Maternal anti-Rh(D) antibodies then bind to fetal RBCs, which subjects them to antibody-dependent lysis. The clinical outcome is variable and ranges from mild anemia to fetal or neonatal death. Before the advent of preventive therapy, clinically significant HDFN occurred in ∼16% of gestations with maternal/fetal Rh incompatibility, or 1 in 1,000 total pregnancies, attesting to the pathogenicity of the response (Tovey, 1992; Urbaniak and Greiss, 2000). Although mice lack Rh(D), a mouse model of Rh disease was developed by Stowell et al. (2013), who expressed the human RBC KEL antigen on fetal RBCs that were otherwise syngeneic to the mother. Antigenic quantities of fetal blood cells enter the maternal circulation of mice only during delivery, and thus anti-KEL antibodies become measurable in the postpartum period and elicit a disease resembling HDFN in subsequent KEL^+^ gestations (Stowell et al., 2013).

Like Rh(D) antigen, paternal HLA molecules can also trigger maternal B cell responses. Anti-paternal HLA antibodies directed against the full set of paternal HLA molecules (HLA-A, -B, -C, and -DR) are identified in most multiparous women and are sometimes detectable in maternal plasma during the third trimester of a first pregnancy. This suggests that, in analogous fashion to the response to Rh(D), the anti-HLA response is driven by exposure to antigenic quantities of blood-borne, HLA-expressing fetal white blood cells as the result of placental microhemorrhage and/or delivery (Honger et al., 2013; Regan et al., 1991; Van Rood et al., 1958). It is also possible that paternal HLA molecules reach the maternal circulation via the transport of fetal cell–derived exosomes across the placenta. However, unlike anti-Rh(D) antibodies, anti-HLA antibodies do not overtly compromise fetal or placental health. Currently, we have no obvious explanation for why this is the case, especially since the same anti-HLA antibodies can mediate transfusion reactions and compromise organ transplantation in multiparous women (Durgam et al., 2022; Porrett, 2018). An intriguing possibility raised by older literature is that placental HLA molecules, either the restricted set expressed by trophoblasts or the more extensive set expressed by placental fibroblasts and macrophages that populate the villous stroma, serve as an “immunoabsorbent” that prevents these antibodies from reaching the fetal circulation (Wegmann, 1981). Specifically, it was found that anti-paternal HLA antibodies could be detected in human placental eluents but not in fetal umbilical cord blood (Doughty and Gelsthorpe, 1974; Tongio et al., 1975; Tongio et al., 1983), and that injection of pregnant mice with radiolabeled allospecific anti–H-2 antibodies, as well as Fab fragments derived therefrom, accumulated in placentas expressing the H-2 target (Wegmann et al., 1979a; Wegmann et al., 1979b). Immunoabsorption per se, of course, does not explain why the antibodies do not damage placental cells (and as we discuss below, perhaps they sometimes do), but we note that both syncytiotrophoblasts in humans and the analogous cells in mice express high levels of regulator proteins that prevent local complement activation (Girardi et al., 2006; Tedesco et al., 1993; Xu et al., 2000).

Somewhat surprisingly, the nature of the maternal CD4 T cell response to Rh(D) and fetal HLA antigens has not been thoroughly addressed. Because maternal anti-Rh(D) and anti-HLA antibodies are class-switched, it has been presumed that maternal CD4 helper T cell responses are also mounted to Rh(D) or a physically associated RBC antigen (since Rh(D) is a component of an integral membrane protein complex). Such CD4 T cell responses are also consistent with the correlation between the number of paternal HLA-derived epitopes predicted to be available to stimulate CD4 T cells during pregnancy and the probability of generating anti-HLA antibodies (Geneugelijk et al., 2015). Moreover, CD8 T cells with reactivity towards paternal class I HLA have been detected in women with anti-paternal HLA antibodies (Bouma et al., 1996), suggesting the generation of T cell help that supports both CD8 T cell and B cell responses. In contrast, recent work showing that T cell help is dispensable for the generation of IgG antibodies to KEL antigen suggests that CD4 T cell–independent, class-switched responses may also occur (Mener et al., 2018).

## Maternal B cell responses to trophoblast antigens

Considering the grave pathogenicity of maternal B cells with specificity toward fetal RBC antigens, the field naturally wondered about the nature of B cell responses to trophoblast-expressed antigens. Experiments performed around 1980 uncovered the presence of “noncytotoxic” anti-paternal IgG1 antibodies in multiparous mice, but closer examination revealed that they arose only in some strain mating combinations and only after multiple gestations (Bell and Billington, 1980; Bell and Billington, 1981). Similar, noncytotoxic anti-paternal IgG1 antibodies could be generated by vaccinating virgin females with crude placental but not fetal extracts (which instead induced a cytotoxic antibody response; Bell and Billington, 1986), suggesting that the IgG1 antibodies might be specific for trophoblast antigens. Along these lines, recently work by Suah et al. (2021) revealed the de novo production of antibodies reactive against paternal-strain spleen cells in the latter half of a first pregnancy in mice. As with the older work, levels of these antibodies were variable and even undetectable in some mice, and all responses were lower than those induced by skin grafting (Suah et al., 2021). Moreover, germinal center phenotype B cells were not detected, suggesting that the antibodies were produced by extrafollicular plasma cells, which canonically yield antibodies of lower affinity. Nonetheless, since mice do not mount responses to fetal RBCs until after delivery (as described above), the antigenic target cell in this case was most likely a trophoblast.

The behavior of maternal B cells with specificity toward a paternal alloantigen known to be expressed by trophoblasts was first directly examined in experiments using female mice bearing BCR transgenes encoding an IgM molecule with relative high affinity for a paternal strain H-2K (Ait-Azzouzene et al., 2001; Ait-Azzouzene et al., 1998). Although results should be interpreted with caution because of the supraphysiologic precursor frequency of monoclonal cells with such a relatively high affinity receptor, a 60–70% deletion of transgenic B cells was observed in maternal spleen, blood, and bone marrow at midgestation in antigen-matched matings but not in syngeneic or third-party matings (Ait-Azzouzene et al., 1998). Interestingly, when expression of the allo–H-2K molecule was restricted to trophoblast giant cells, deletion was observed for developing bone marrow B cells but not peripheral B cells (Ait-Azzouzene et al., 2001). Together, these findings suggest that cells expressing a BCR with high affinity for trophoblast antigen might be subject to deletion in the bone marrow, an obvious safeguard against the generation of high-affinity anti-trophoblast antibodies.

Our laboratory has recently examined the fate of the endogenous repertoire of peripheral B cells specific for a trophoblast antigen (Rizzuto et al., 2022). This work used a pregnancy model previously used to study T cell responses in which a paternally inherited transgene directs expression of a transmembrane form of chicken OVA (mOVA; Ehst et al., 2003) throughout the conceptus, with particularly high expression by trophoblasts positioned within the maternal bloodstream (Erlebacher et al., 2007). By using fluorescently labeled antigen tetramers to visualize OVA-specific B cells in the spleen (Taylor et al., 2012), we found that such B cells sensed trophoblast mOVA (t-mOVA) starting around midgestation, when the antigen starts being shed into the maternal circulation. Even in the presence of systemic adjuvant and T cell help, the OVA-specific B cells failed to expand and did not differentiate into germinal center cells. Strikingly, mOVA mating also rendered OVA-specific B cells unable to respond to vaccination with chicken-derived OVA (c-OVA), demonstrating that t-mOVA induced the suppression of antigen-specific B cells.

Because the protein coding sequence of c-OVA and trophoblast-derived OVA is identical, we evaluated whether a trophoblast-specific posttranslational modification could explain the divergent response of B cells to the two forms of the protein. Indeed, while both sources of OVA were decorated with *N*-linked glycans, a biochemical analysis revealed that t-OVA was more heavily glycosylated and that its glycans were terminated with α2,6-linked and α2,3-linked sialic acid residues (Rizzuto et al., 2022). These sialic acids can serve as “self” ligands by binding to sialic acid–binding I_g_-like lectins (Siglecs), a family of transmembrane proteins that typically inhibit immune cell activation (Laubli and Varki, 2020). Accordingly, we found that OVA-specific B cells were no longer suppressed in mice lacking the B cell–specific inhibitory Siglec, CD22, which recognizes α2,6-linked sialic acids, and that the maternal B cell response to t-mOVA was fully unleashed in pregnant mice lacking LYN, the Src family tyrosine kinase member uniquely required for CD22 inhibitory activity (Rizzuto et al., 2022; Smith et al., 1998). That the response to trophoblast antigen was more dramatic in *Lyn*^−/−^ versus *Cd22*^−/−^ pregnant females hints at engagement of additional inhibitory receptor(s) upstream of LYN, such as Siglec-G (Nitschke, 2014).

These observations suggest a critical role for protein glycosylation in suppressing antigen-specific maternal B cell responses to trophoblast antigens. Intriguingly, this idea was foreshadowed by both an old hypothesis stating that trophoblast antigens are masked by a “sialomucin coat” (Beer and Sio, 1982), and by experiments in the 1960s, which, although controversial at the time, showed that neuraminidase treatment unmasks immunogenicity of crude mouse placenta homogenates (Currie et al., 1968; Taylor et al., 1979). While sialylated glycans decorate most mammalian cell surface proteins, an identical protein that is expressed in different cell types (or by the same cell type under various conditions) is often differently glycosylated because of the cells’ differential expression of sugar transporters, glycosyltransferases, or deglycosylases or as the result of dissimilar transit speeds through the ER/Golgi processing pathway (Varki et al., 2022). Accordingly, as compared with mOVA expressed by nonplacental organs such as the adult skin (where mOVA serves as a rejection antigen; Ehst et al., 2003), mOVA in maternal plasma was more sialylated as well as more glycosylated in general (Rizzuto et al., 2022). Moreover, its levels of α2,6-sialylation and overall glycosylation matched that of mOVA from placental tissue but not the fetus proper, confirming that its source was indeed the placenta, where, as mentioned, the protein is expressed at particularly high levels by trophoblasts bathed in maternal blood. We also found that glycoproteins of known or likely trophoblast origin bearing α2,6-linked sialic acids are present in pregnant plasma of mice and humans, indicating that relevant sialylation pathways also apply to bona fide trophoblast antigens. Differential glycosylation, including the extent of sialylation, thus provides an explanation for how antigens expressed by trophoblasts can be uniquely tolerogenic. Indeed, we suggest that differential glycosylation of fetal nontrophoblast versus trophoblast proteins could at least in part explain the differences in maternal B cell reactivity toward fetal blood antigens versus trophoblast antigens (Fig. 1). Consistent with this idea, Rh(D) antigen appears to be entirely devoid of associated glycans, unlike nearly all other mammalian cell surface proteins (Gahmberg, 1983; Moore and Green, 1987).

## Maternal T cell responses to trophoblast antigens

Experiments in mice, again largely using the mOVA system described above, have revealed several distinct mechanisms that limit maternal CD4 T cell responses to trophoblast antigens. One mechanism documented for early gestation is the entrapment of migratory dendritic cells (DCs) within the decidua, which precludes input from these potent APCs in the uterine draining lymph nodes (Collins et al., 2009). Instead, t-mOVA undergoes cell-free transport within myometrial lymphatic vessels, akin to its cell-free transport within the maternal bloodstream described above. Accordingly, the antigen is taken up and presented by spleen- and lymph node–resident APCs. Since these APCs are of maternal origin, maternal T cell awareness of the fetoplacental allograft thus occurs entirely via the “indirect” allorecognition pathway and not the “direct” or “semidirect” pathways that would involve, respectively, antigen presentation by fetal APCs or the transfer of intact fetal MHC–peptide complexes to maternal APCs (Erlebacher et al., 2007). Sole reliance on the indirect allorecognition pathway might itself be considered a mechanism of fetomaternal tolerance, since it substantially reduces the number of T cells participating in the rejection response. However, indirect allorecognition is sufficient to trigger organ rejection (Marino et al., 2016), and in fact expression of the mOVA antigen alone is sufficient to elicit the rejection of otherwise syngeneic skin grafts (Ehst et al., 2003). Thus, an outstanding question has been the exact nature of maternal T cell responses to trophoblast antigens and how they might differ from responses to pathogen-encoded antigens or minor histocompatibility antigens encountered following organ transplantation, which are similarly presented by the indirect allorecognition pathway.

The fate of trophoblast antigen-specific CD4 T cells has been investigated using the mOVA system in conjunction with T cell adoptive transfers, as well as a modified version of this system in which a model antigenic peptide, 2W1S (Rees et al., 1999), is incorporated into mOVA construct (generating 2W1S-mOVA). This latter approach allows for the visualization of the endogenous repertoire of CD4 T cells using 2W1S-MHCII tetramers. Perhaps not surprisingly, given that pregnancy is not an inflammatory state that induces the systemic activation of maternal APCs, CD4 T cells responding to t-mOVA or trophoblast 2W1S do not differentiate into T helper 1 (T_H_1) cells (Rizzuto et al., 2022; Rowe et al., 2012). However, OVA-specific CD4 T cells fail to differentiate into T_H_1 cells even when the mice are given adjuvants, and OVA- and 2W1S-specific CD4 T cells both fail to differentiate into T_H_1 cells when the mice are respectively injected with c-OVA plus adjuvants or infected with 2W1S-expressing *Listeria monocytogenes*, two manipulations that generate strong T_H_1 responses when mice do not bear mOVA^+^ concepti (Rizzuto et al., 2022; Rowe et al., 2012). These observations indicate that trophoblast antigen-specific CD4 T cells experience dominant immune suppression, and the field has largely focused on the potential role of regulatory T cells in mediating this suppression. Accordingly, 2W1S-specific regulatory T cells (Tregs) expand concurrently with the release of 2W1S-mOVA antigen from the placenta beginning at midgestation (Kalekar et al., 2016; Rowe et al., 2012; Suah et al., 2021), and partial depletion of these cells disinhibits IFNγ production by the non-converted population of 2W1S-specific CD4 T cells (Rowe et al., 2012). The idea that Tregs are important for pregnancy is also supported by observations that systemic Treg cell frequencies increase at midgestation, and more so in allogeneic than syngeneic pregnancies (Aluvihare et al., 2004; Rowe et al., 2011; Thuere et al., 2007; Zhao et al., 2007). In addition, partial Treg cell depletion beginning at midgestation, achieved by administration of diphtheria toxin to mice that express the diphteria toxin receptor from the *Foxp3* locus, results in a significant rate of fetal loss following allogeneic but not syngeneic mating (Chaturvedi et al., 2015; Rowe et al., 2012). A similar, but lesser degree of fetal loss is evident in mice lacking the *Foxp3* locus enhancer element called conserved noncoding sequence 1 that is required for peripheral conversion of naive CD4 T cells to induced Tregs (Samstein et al., 2012). Provocatively, the conserved noncoding sequence 1 element is uniquely found within placental mammals (Andersen et al., 2012), thus linking the evolution of the placenta with placental-specific induced Tregs that may foster tolerance to trophoblast antigens by suppressing effector T cell responses.

Surprisingly, our recent work on trophoblast glycans revealed that OVA-specific CD4 T cells are not disinhibited when Tregs are depleted during mOVA pregnancies, even though these pregnancies cause OVA-specific CD4 T cells to convert to Treg cells to some extent (Rizzuto et al., 2022). We also unexpectedly found that immunodominant t-mOVA peptides are presented to splenic CD4 T cells by OVA-specific B cells, and not DCs. Such B cell–exclusive antigen presentation, which we speculate is a consequence of how trophoblast antigen glycans influence antigen transport within the spleen, might thus also be considered a mechanism of fetomaternal tolerance, since strong CD4 T cell responses usually require input from DCs (Archambault et al., 2013). Moreover, we found that the glycan-mediated suppression of the OVA-specific B cells contributes to the suppression of OVA-specific CD4 T cells, since the CD4 T cell suppression is partially reversed in pregnant LYN-deficient mice whose B cells can no longer signal via inhibitory Siglecs, and which show robust B cell responses to t-mOVA (Rizzuto et al., 2022). It is likely that these results will be relevant to CD4 T cell responses to the 2W1S antigen, since the 2W1S-mOVA protein is only 14 amino acids longer than the mOVA protein and is presumably decorated by trophoblasts with the same immunosuppressive glycans as t-mOVA. The intersection of glycan-suppressed B cells and the generation of antigen-specific Tregs currently remains unclear, although B cell–deficient μMT mice intriguingly show blunted midgestational expansion of maternal Tregs (Busse et al., 2019). Thus, B cell–mediated presentation of trophoblast antigens may explain why trophoblast antigen-specific Tregs expand during pregnancy, but with the ultimate importance of Tregs in maintaining fetomaternal tolerance depending on the specific antigen in question.

In contrast to the more classically tolerance-like maternal CD4 T cell response to trophoblast-derived antigens, the CD8 T cell response is best described as nonimmunogenic, since this response is characterized by neither tolerance nor immunity. Thus, while CD8 T cells do not become activated to t-mOVA, even when the mice are given adjuvants, mOVA-mated mice show robust CD8 T cell responses to OVA vaccination (Tay et al., 2013). Moreover, trophoblast antigen-specific CD8 T cells persist postpartum in an antigen-experienced/quasi-memory state and can participate in the antigen-specific rejection of skin grafts and tumor cells, albeit with reduced effector capacity (Barton et al., 2017; Jasti et al., 2017; Kinder et al., 2020; Lewis et al., 2022). It currently remains unclear the extent to which this response can be explained by differences in the APC type that present trophoblast antigens to CD4 and CD8 T cells (B cells versus DCs in the case of t-mOVA; Rizzuto et al., 2022), or whether the sialylation of trophoblast antigens influences the CD8 T cell response.

## Are there any instances of antigen-specific placental rejection?

Although not the topic of this review article, mechanisms also exist to protect the conceptus from attack by T cells, should they happen to become activated, as well as mechanisms that prevent antibodies from damaging trophoblasts. These mechanisms rely on the unique tissue characteristics and immunology of the maternal–fetal interface (reviewed in Erlebacher [2013]), as well as the high expression of complement regulatory proteins by trophoblasts, as alluded to above. Given this redundancy, it is perhaps not surprising that manipulations that induce what we would consider to be true placental rejection have not yet been described. We acknowledge that many perturbations can trigger fetal loss in mice. However, some of these, such as low-dose LPS or systemic maternal B cell and DC activation with anti-CD40 antibodies in the peri-implantation period, induce fetal loss via nonspecific inflammation that does not involve an antigen-specific component and thus cannot be considered ruptures in fetomaternal tolerance (Erlebacher et al., 2004; Gendron et al., 1990). Other manipulations, including maternal Treg cell depletion (Rowe et al., 2011), PD-L1/PD-1 blockade (Guleria et al., 2005), myeloid-derived suppressor cell depletion (Ostrand-Rosenberg et al., 2017), and indoleamine 2,3-dioxygenase inhibition (Munn et al., 1998), show greater loss of allogeneic versus syngeneic concepti and are thus more indicative of antigen-specific rejection. However, in some of these models, the fetal loss was no longer observed in the genetic knockout (Baban et al., 2004; Taglauer et al., 2009), and in all mouse models of fetal loss to date, antigen-specific T cells have not been observed to accumulate in an appreciable way at the maternal–fetal interface. This situation thus raises questions about exact effector mechanisms, since it contrasts with the robust T cell infiltration observed during organ and tumor rejection. Remarkably, several case reports now show that pregnancy is unharmed in women with cancer who received checkpoint blockade inhibitors in early gestation, thus suggesting that these pathways are not singularly required for fetomaternal tolerance (Xu et al., 2019).

A single clinical situation, which like many pregnancy complications is currently of unclear pathogenesis, may in fact represent coordinated T cell– and antibody-mediated attack of the placenta. Villitis of unknown etiology (VUE) is diagnosed when, in the absence of infection, histologic examination of the placenta reveals a lymphocyte infiltrate in the stroma of the trophoblast-lined placental villi (Kim et al., 2015; Redline and Patterson, 1993; Tamblyn et al., 2013). This infiltrate predominantly consists of maternal CD8 T cells, and affected villi show expression of T cell–recruiting chemokines with upregulation of ICAM-1 and the deposition of complement components on a damaged syncytiotrophoblast layer (Ito et al., 2015; Lee et al., 2013; Kim et al., 2008; Kim et al., 2009; Rudzinski et al., 2013). Cases of VUE are designated as mild or severe based on the extent of villus involvement. Mild VUE is focally limited, is not associated with adverse pregnancy outcomes, and is observed in upwards of a third of all mature placentas. Severe VUE, on the other hand, is characterized by diffuse infiltration of placental villi and is associated with a mild increase in the risk of stillbirth and the presence of maternal T cells within the fetus (Redline, 2007). Consistent with an adaptive immune etiology, severe cases show a high recurrence risk in subsequent pregnancies (Redline and Abramowsky, 1985). Moreover, there is an association between VUE and the detection of maternal anti-HLA antibodies in serum (Lee et al., 2011).

Drawing on the divergence in maternal responses to trophoblast versus fetal blood cell antigens outlined above, we propose the following model for VUE pathogenesis that is based on the priming of maternal T and B cells specific for fetal blood cell antigens rather than trophoblast antigens. First, placental microhemorrhage exposes maternal T cells to fetal blood cells that express paternal allogeneic HLA. Maternal CD4 and CD8 T cells are then activated via the direct allorecognition pathway, with CD4 T cells also providing help for a B cell response. Second, there is focal damage to the usually impenetrable syncytiotrophoblast layer, perhaps via alloantibody binding and complement activation, and potentially involving the killing of trophoblasts. This allows CD8 T cells to gain access to the underlying villous stroma, where they encounter fetal fibroblasts, macrophages, and endothelial cells, which express the full set of paternal class I HLA, thus vigorously activating the T cells and reinforcing the inflammatory reaction. It is also possible that T cells gain entry to the villous stroma in focal areas where the syncytiotrophoblast layer has been damaged by a pathogen, or merely by compromised blood flow.

## Summary and future prospects

Now is a very exciting time in reproductive immunology, as newly developed tools are allowing us to thoroughly dissect adaptive immune responses to the fetoplacental allograft. Based on the current literature, we have tried here to articulate a framework that explains why these responses appear to be so divergent, with trophoblast antigens inducing tolerance and fetal blood cell antigens driving immunity (Fig. 1). This framework relies on clinical observation and the study of a limited number of “model” antigens in mice (such as mOVA, 2W1S-mOVA, and KEL), and thus an important next step is to determine the extent to which the same principles we articulate here apply to bona fide trophoblasts antigens, as well as to fetal proteins that are transported across the placenta into the maternal bloodstream. It is also unclear the extent to which these principles apply to CD8 T cell responses to fetal and placental antigens. Indeed, many minor histocompatibility antigens, including those encoded by the Y-chromosome and that appear to elicit maternal CD8 T cell responses (James et al., 2003; Lissauer et al., 2012; van Kampen et al., 2001; Verdijk et al., 2004), show both placental and fetal expression, and it is unclear whether they reflect exposure to trophoblasts versus fetal blood cells. It will also be crucial to determine the extent to which the maternal immune system is even aware of the non–plasma membrane proteins of trophoblasts, noting that cytoplasmic proteins are much less likely to be modified with glycans of any kind.

Mechanisms of increased trophoblast membrane protein sialylation are also an open area of research. As alluded to above, these mechanisms might include increased activity of sialyltransferases and sialic acid transporters, decreased activity of sialidases, and altered transit through the ER/Golgi processing pathway. Trophoblast antigen sialylation (and glycosylation more generally) might also be regulated in subtype-specific fashion and under the influence of paracrine factors. Indeed, recent evidence suggests that the glycan pattern of extravillous trophoblasts located in the uterine decidua might in part be regulated by cross talk with uterine natural killer cells and DCs (Borowski et al., 2020). There may also be important, perhaps hormonally driven, changes in the glycosylation of maternally expressed proteins during pregnancy. For example, maternal IgG species are well documented to become more galactosylated and sialylated during pregnancy, and the extent of this increase shows a striking association with the amelioration of rheumatoid arthritis that is often observed in latter gestation (Bondt et al., 2018; van de Geijn et al., 2009). Lastly, we note that patients with lupus, an autoimmune disease characterized by B cell hyperactivity, show decreased protein levels of LYN (Brodie et al., 2018) and high rates of pregnancy complications (Buyon et al., 2015). These observations raise the possibility that changes in the glycosylation of trophoblast antigens, or in how maternal immune cells respond to trophoblast antigen-associated glycans, can cause these antigens to become pathologically immunogenic in some patients.
